# Genetic Deficiency in Neprilysin or Its Pharmacological Inhibition Initiate Excessive Stress-Induced Alcohol Consumption in Mice

**DOI:** 10.1371/journal.pone.0050187

**Published:** 2012-11-21

**Authors:** Björn Maul, Matthias Becker, Florian Gembardt, Axel Becker, Heinz-Peter Schultheiss, Wolf-Eberhard Siems, Thomas Walther

**Affiliations:** 1 Department for Biochemical Neurobiology, Leibnizinstitut für Molekulare Pharmakologie, Berlin, Germany; 2 Department of Cardiac Pathobiology, Excellence Cluster Cardio-Pulmonary System, Giessen, Germany; 3 Department for Nephrology – MK3, University Hospital Dresden, Dresden, Germany; 4 Institute of Pharmacology and Toxicology, Otto-von-Guericke-University of Magdeburg, Magdeburg, Germany; 5 Department Cardiology, Charité – Campus Benjamin Franklin, Berlin, Germany; 6 Institute for Experimental and Clinical Pharmacology and Toxicology, Medical Faculty Mannheim – University Heidelberg, Mannheim, Germany; 7 Centre for Fetal Medicine, Department of Pediatric Surgery & Department of Obstetrics, Division of Women and Child Health, University of Leipzig, Leipzig, Germany; Nathan Kline Institute for Psychiatric Research and New York School of Medicine, United States of America

## Abstract

Both acquired and inherited genetic factors contribute to excessive alcohol consumption and the corresponding development of addiction. Here we show that the genetic deficiency in neprilysin [NEP] did not change the kinetics of alcohol degradation but led to an increase in alcohol intake in mice in a 2-bottle-free-choice paradigm after one single stress stimulus (intruder). A repetition of such stress led to an irreversible elevated alcohol consumption. This phenomenon could be also observed in wild-type mice receiving an orally active NEP inhibitor. We therefore elucidated the stress behavior in NEP-deficient mice. In an Elevated Plus Maze, NEP knockouts crossed more often the area between the arms, implicating a significant stronger stress response. Furthermore, such animals showed a decreased locomotor activity under intense light in a locomotor activity test, identifying such mice to be more responsive in aversive situations than their wild-type controls. Since the reduction in NEP activity itself does not lead to significant signs of an altered alcohol preference in mice but requires an environmental stimulus, our findings build a bridge between stress components and genetic factors in the development of alcoholism. Therefore, targeting NEP activity might be a very attractive approach for the treatment of alcohol abuse in a society with increasing social and financial stress.

## Introduction

Alcoholism is a most devastating disease affecting broad parts of the western society. In the US alone, 14 million people meet standard criteria for alcohol abuse or alcoholism [1], triggering massive expenses in health care (approximately $180 billion dollars per year). The devastating disease process often causes enormous harm to the patients, their family members and their social environment. Alcohol abuse is a prototypic complex disease which is determined jointly by multiple genes and environmental influences [2]. Numerous studies have found that stress increases alcohol consumption in animals and humans [3], [4]. Individual differences are based on both environmental influences and genetic factors [5]. Studies in genetically modified animals or with distinct pharmacological interventions described that individual alcohol consumption is influenced by endogenous peptidergic systems [6]. Our own experiments focusing on the renin-angiotensin system (RAS) confirm this concept [7], [8]. Interestingly, numerous peptides involved in these motivational procedures (e.g. opioids, CRH, angiotensin) are enzymatically degradable by a small group of peptidases, including neprilysin (NEP; neutral endopeptidase, EC.3.4.24.11). NEP is a widely distributed transmembranal metallopeptidase with a broad spectrum of endogenous substrates and being associated with human diseases like Alzheimer disease [9] and obesity [10]. Therefore, we wanted to investigate in detail the effect of the lack of NEP activity on voluntary alcohol consumption.

## Results and Discussion

**Figure 1:**
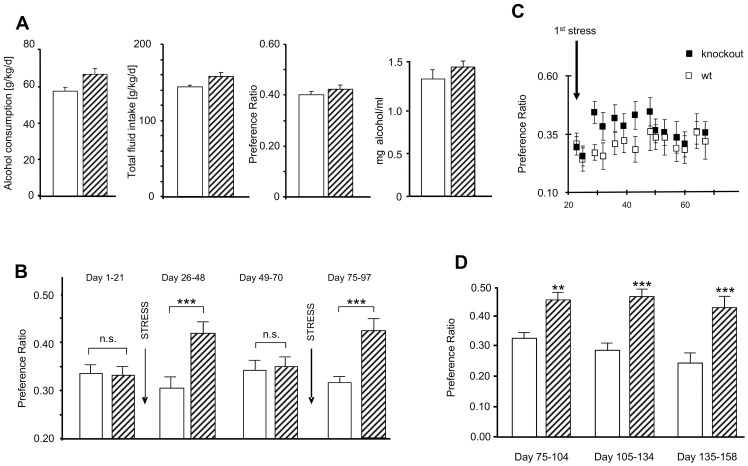
Influence of NEP activity on the alcohol-preference ratio in transgenic mice. (**A**) No significant differences between NEP-deficient (hatched bars) and NEP wild-type mice (white bars) in absolute alcohol consumption (left panel), total fluid consumption (second left panel), alcohol/total fluid ratio (second right panel), and in alcohol degradation (measured 2 h after alcohol i.p. injection; right panel) under stress-free conditions; (**B**) Development of stress-induced differences in the preference ratio between the both genotypes; (**C**) Development of stress-induced differences in the preference ratio between the both genotypes between days 22 and 70 of the experiment; (**D**) No fading of stress-induced differences after second stress phase in the preference ratio of NEP-deficient and NEP wild-type mice at 3 consecutive periods of approx 30 days. All data sets include at least measurements at 8 independent successive time points. **P<0.01, ***P<0.001 vs. wild-type.

The catabolic action of NEP on several peptides involved in individual alcohol intake (e.g. opioids, angiotensin II, substance P) led us to investigate the impact of its deficiency on alcohol consumption. While we primarily identified such NEP-deficient animals as mice with significantly more alcohol intake [11], a repetition of this approach in new animal facilities could only demonstrate a tendency for an increased alcohol intake in such mice having free access to alcohol for 4 weeks in comparison to their age-and gender-matched wild-type controls (**Figure 1A**, left panel). Taking the slight increase in total fluid consumption (**Figure 1A**, second left panel) into consideration, such trend in alcohol intake has been almost blunted when the alcohol/total fluid ratio was calculated (Figure 1A, second right panel).

Importantly, we could also show in an independent set of animals that the kinetics of alcohol degradation was not different between NEP-deficient mice and their wild-type controls (**Figure 1A**, right panel).

The only difference between both animal facilities were possible stress components within the first round of experiments due to livestock keeping of animals in a frequently used open-plan room and an experimental setting using groups of 4–5 animals. In the second setting however, the experiments were performed in a separate experimental room and constructed by 2 animals per cage separated by a perforated plexiglas wall, avoiding the development of social stress. Since NEP also degrades peptides being involved in stress-motivated drinking (e.g. CRH; [4]), the authors therefore hypothesized that NEP is a strong candidate for the elusive genetic factor in the relationship between stress and alcoholism.

Therefore, we carefully arranged two-bottle-free-choice experiments under stress-free conditions and under social stress conditions in male NEP-deficient mice (n = 22) and corresponding wild-type controls (n = 16). Four-month-old animals were observed for 21 days and as before no difference in alcohol intake (10% alcohol) between both genotypes could be identified under such new conditions avoiding the development of stress. On day 22, animals were stressed by intruder as described in detail by Sillaber and colleagues [4]. As shown in **Figure 1B**, NEP-deficient males drank significantly more alcohol after stress, while the wild-types did not show increase in alcohol consumption (day 26–48). Notably, such increase occurred very fast since the second measurement after stressing the mice showed already the significantly increased alcohol consumption in the NEP knockouts (**Figure 1C**). However, approx. 3 weeks after stress, NEP deficient animals returned back to normal. After such normalization phase (day 49–70), the animals were re-stressed with the same stress method. As described above for the first intruder-mediated stress, NEP–deficient mice drank significantly more alcohol (day 75–97). However, in contrast to the first stress, there was no fading and NEP-deficient mice remained drinking significantly more alcohol until the end of the experiment (day 158) (**Figure 1D**).

Although the number of candidate peptides affected by NEP alteration is significant, we started already first investigations showing e.g. lower enkephalin degradation in a variety of brain regions of NEP-deficient mice. Such higher enkephalin levels, however, did not influence the alcohol consumption under basal (unstressed conditions) (data not shown). Thus, still lacking a mechanistic explanation for such finding, the alcohol data might illustrate the decisive role of NEP for alcohol preference in mice and probably also for alcoholism in men.

To evaluate whether the well-documented relation between stress sensitivity and increased alcohol intake in men could also be the basis for our findings in NEP-deficient mice, we tested the hypothesis that NEP-deficient animals might have a different stress response and therefore a higher alcohol preference. Both genetic lines were tested in two independent behavioral paradigms.

**Figure 2:**
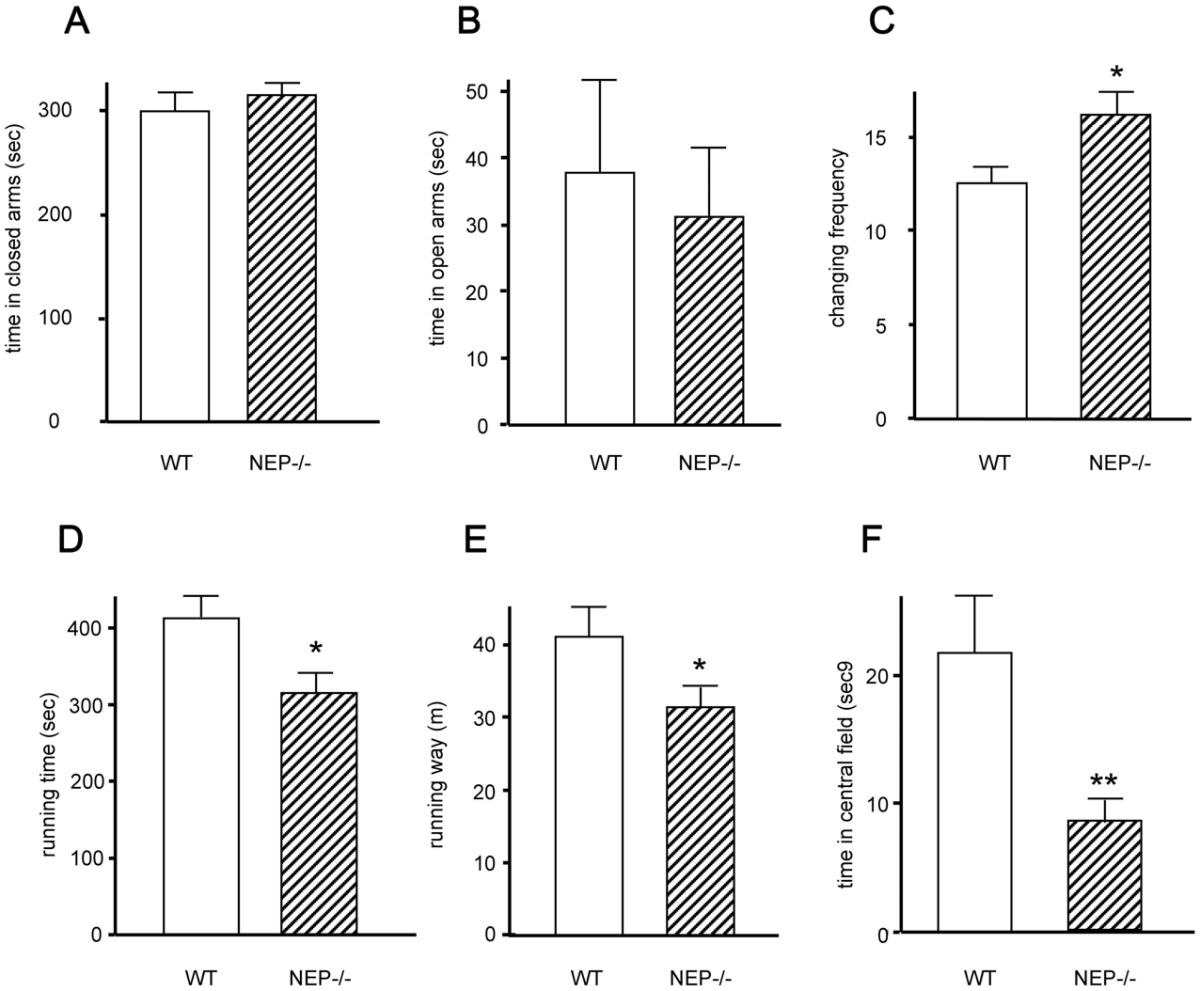
Response of mice with NEP deficiency in two independent tests of emotional behavior. NEP deficient mice (hatched bars) did not show alterations in the time spent in the (**A**) closed arm and (**B**) in the time travelling, (**C**) but number of transitions between the chambers crossed the area between the arms significantly more frequent. (**D**) Using a motility monitor system, NEP-deficient mice did show significant reduction in travelling time, (**E**) distance travelled, and (**F**) time in the middle (open field) of the monitor system. All data sets include at least measurements at 8 independent successive time points. *P<0.05, **P<0.01 vs. control.

In the EPM, total number of arm entries and number of closed-arm entries are usually considered as measures of general activity [12]. Its reduction and a longer time in the closed arm are interpreted as anxious behavior. However, the NEP-knockout males did not show any alteration for such parameters implicating no increase in anxiety in mice with NEP deficiency (**Figure 2A and B**). On the other hand, an overwhelming urge to escape from an environment or situation can be an important feature of panic disorder [13]. In line with this, Blanchard and colleagues [14] have proposed that this pattern of behavior shown by mice in threatening contexts may be analogous to the behavioral symptoms of panic. The EPM test is basing on a conflict between exploration and aversion to elevated open places [15]. As shown in **Figure 2C**, NEP-deficient animals crossed significantly more often the area between the arms, implicating that these animals are more responsive in unfamiliar situations and thus show a significant stronger stress response.

To further evaluate the stress response of the NEP-deficient mice, a locomotor activity test was performed to measure further components of emotional behavior. The test was performed under different illumination levels (30 lux vs. 450 lux). It is well-known that the conditions of illumination can dramatically alter the response to a novel environment [16], [17], whereby intense light conditions are considered to stimulate aversion in rodents [12]. When tested under low-light conditions (30 lux), there were no differences between NEP-deficient mice and their wild-type controls in all parameters measured in this test, indicating no differences in the emotional state under less aversive conditions (data not shown). In contrast, when the Moti-test was performed under intense light conditions (450 lux), locomotor activity (in terms of distance and time travelled) and percent of time spent in the centre of the test box (which reflects components of emotional behavior) were significantly decreased in NEP-knockout mice (**Figure 2D–F**). This identifies such mice to be more responsive in aversive situations, confirming the EPM data that NEP deficiency leads to a stronger behavioral response under stress conditions.

**Figure 3:**
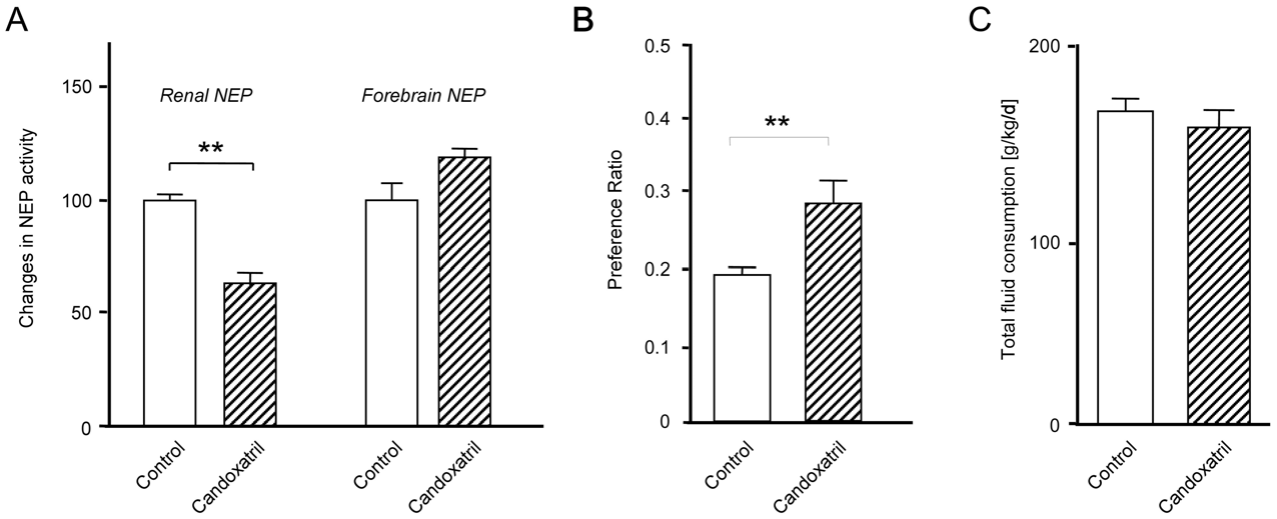
Influence of pharmacological inhibition of NEP activity on the alcohol-preference ratio. (**A**) Effects of candoxatril (200 mg/kg/day) on central and peripheral NEP activity; (**B**) Candoxatril-induced differences in the alcohol preference ratio and (**C**) total fluid intake. All data sets include at least measurements at 8 independent successive time points. **P<0.01, ***P<0.001 vs. control.

To test whether the stress-mediated increase in alcohol consumption requires life-long NEP deficiency as in the genetically deficient NEP-knockout mice, we used a pharmacological approach in an independent experimental set of wild-type mice. Four-month-old animals were fed either with standard diet or food pellets with 200 mg/kg/day candoxatril (each group n = 8), a NEP-specific inhibitor, as a food additive. Under such conditions, candoxatril exclusively reduced the peripheral NEP activity (**Figure 3A**). Notably, other peptidases like ACE and ECE were not altered in their activity under candoxatril treatment (data not shown). Fourteen days after treatment start, animals received 10% alcohol or water in a two-bottle-free-choice experiment. In contrast to the approach with NEP knockouts (2 animals per cage separated by a perforated plexiglas wall), social stress was not avoided (no separation) in the candoxatril experiment. As shown in **Figure 3B**, also the pharmacological NEP inhibition resulted in a highly significant increase in alcohol consumption. Notably, the increase in the preference ratio related only to more alcohol intake while the general fluid consumption was not influenced by candoxatril (**Figure 3C**).

Thus, taken together our data shows that the deficiency in NEP and the pharmacological inhibition of this peptidase lead to a stress-induced increase in alcohol intake in a 2-bottle-free-choice paradigm. Nowadays, a variety of pharmaceutical companies developed NEP inhibitors for the treatment of cardiovascular diseases [18], [19]. However, our data implicate that their use might have a significant side effect for people under social stress or with financial problems, since the NEP inhibition in such individuals might stimulate an alcohol abuse.

Our findings are all the more important, since we used candoxatril, a NEP inhibitor unable to pass the blood-brain barrier, illustrating the crucial role of peripheral NEP for alcohol preference. Recently, a variety of authors have suggested the existence of a gut-brain axis, e.g. demonstrating the impact of signaling initiated by stimuli in the gut on exteroceptively generated emotions [20]. Therefore, there are substantial mechanistic explanations for our findings that peripheral accumulation of endogenous NEP substrates is regulating alcohol consumption. Although it is still speculative which peptides, NEP is decisive for their generation or degradation, are responsible for the observed effects in mice lacking NEP-activity, we could already demonstrate in previous studies that NEP deficiency modifies the concentration of endogenous peptides like GLP-1, NPY and galanin [21], which has been demonstrated to influence behavior [22]. Therefore, a better understanding of the molecular mechanisms involved in such gut-brain cross-talk for the motivation to voluntarily drink alcohol is an important prerequisite for the development of new therapies. Although a link between stress and alcohol is well accepted, there are only a very few papers showing a strict genetic determinant that is involved in that interrelation. Since the absence of NEP activity itself does not lead to signs of alcohol preference in mice but requires an environmental stimulus, our findings build a bridge between stress components and genetic factors in the development of alcoholism. Therefore, stimulating NEP activity might be a very attractive approach for the treatment of alcohol abuse.

## Materials and Methods

### Animals

We used male NEP-knockout mice that were originally generated by Lu [23] and maintained in the breeding stocks of T.W. at the Charité, Campus Benjamin Franklin (CBF), Berlin, Germany [10], [21]. Homozygous NEP-knockout mice and wild-type controls were bred from parents, which were F2 after hemizygous mating and being on a C57Bl/6N background. Animals were housed in litters separated according to sex at 22±1°C in a 12 h/12 h light/dark cycle with unrestricted access to food and water. Recordings of physiological parameters were performed between 9:00 a.m. and 1:00 p.m.

All experiments were performed under the regulation and permission of the Animal Care Committee of the Erasmus MC, Rotterdam, The Netherlands and the local authorities in Germany (Landesamt für Gesundheit und Soziales des Landes Berlin). The investigation conforms to the *Guide for the Care and Use of Laboratory Animals* published by the US National Institutes of Health (NIH Publication No. 85–23, revised 1996).

### Preference test in two-bottle-free-choice paradigm

Experimental animals were kept in groups of two animals per cage. Each cage was divided into two equal compartments by a Plexiglas divider as we have described previously [7]. The mice were held in a free-choice paradigm with a bottle of tap water and a bottle containing a 10% (v/v) ethanol solution. Food and beverage were available *ad libitum*. Water and alcohol consumption were recorded every third day.

### Two-bottle-free-choice paradigm under social stress

Social stress experiments were performed as previously described [4], [24]. In brief, single experimental animals have been set into a cage with three unfamiliar age-matched male mice, which have been together in their cage for at least 1 week. The new animal has been immediately attacked by the home-cage mice (less than a minute). Directly after such physical contact, the experimental mouse has been separated from the group within the cage by introducing a Plexiglas wall between the 3 home-cage mice and the single mouse. Such visual contact has been kept for 15 min. Then, the experimental mouse has been transferred back to its own home cage and the two-bottle-free choice experiment has been continued.

### Treatment with candoxatril

Standard food (“ssniff SM/R/N-H (10 mm)”; ssniff Spezialdiäten GmbH, Soest, Germany) was cold milled, supplemented with the frequently used, well tolerated, orally active and tasteless NEP-inhibitor candoxatril (Pfizer Deutschland GmbH, Karlsruhe, Germany) [25], [26], mixed and grouted under pressure (2 g/kg); this corresponds to a daily consumption of approx. 200 mg/kg/day, a dose that has been identified in dose-finding experiments [10]. The manufacturing of candoxatril-containing food was performed as usually by ssniff Spezialdiäten GmbH. The used control food was prepared at the same day by the same management [10].

### Kinetics of alcohol degradation

The measurement of blood alcohol concentration after oral application of 10% alcohol was performed using the microphotometer LP20, according to the manufacturer's manual (LZC32, 2000) of Dr. Lange GmbH & Co D-14163 Berlin (Germany).

### Measurement of NEP activity

NEP activity was measured in kidney and forebrain homogenates on the basis of a general method described by Winkler [27]. [D-Ala^2^, Leu^5^] enkephalin (DALEK, 200 μM) in a 50 mM Tris-buffer (pH 7.4) was used as substrate. To prevent the degradation of DALEK by ACE and by aminopeptidases, lisinopril (10^−6^ M) and bestatin (10^−4^ M) were added. The reaction was performed in a thermo-shaker at 37°C, and stopped by addition of 0.35 M perchloric acid (HClO_4_), centrifuged (5.000 g), and the supernatants stored till HPLC-analysis at 4°C. The resulting degradation product Tyr–D-Ala–Gly (TAG), as well as the remaining DALEK concentration, were measured by high performance liquid chromatography (HPLC) using a RP-C18 column and an isocratic fluid phase consisting of an acid perchlorate-phosphate-buffer containing 6% acetonitrile. The peptide peaks as well as standard concentrations of TAG and DALEK were detected at 216 nm (UV). The NEP-specificity of the reaction was characterized in parallel assays using the NEP inhibitor 10^−5^ M candoxatrilat (UK73,967; Pfizer, Karlsruhe, Germany).

### ACE activity

ACE activity was measured with a fluorimetric method using Hip-His-Leu as substrate and His-Leu as standard reagent as described previously [24]. Fluorescence arising from His-Leu after reaction with o-phthalaldehyde was measured at 365 nm (excitation) and 500 nm (emission). The activity is expressed as nmol His-Leu/min/mg protein.

### ECE activity

ECE-1 activity was measured as described in detail by Johnson and Ahn [28] using the DNP-quenched fluorogenic substrate Mca-Arg-Pro-Pro-Gly-Phe-Ser-Ala-Phe-Lys-DNP.

### [Leu^5^]enkephalin degradation

[Leu^5^]enkephalin degradation by membrane preparations was investigated as previously described [11]. In brief, 100 µM [Leu^5^]enkephalin in 50 mM Tris-buffer (pH 7.4) was incubated with membrane preparations of a final protein concentration of 0.5 mg/ml. The reaction was stopped by addition of 0.35 M HClO_4_. The remaining [Leu^5^]enkephalin concentration was quantified by HPLC (Shimadzu, isocratic gradient of acetonitril [25%] and 75% NaClO_4_ [0.15 M]/NaH_2_PO_4_ [0.01 M] buffer [pH 2.2], nucleosil column [100 C18]) at 216 nm.

### Elevated plus maze

Fear was measured in an elevated plus maze (EPM). The maze was made of black polyvinyl chloride and had two open and two closed arms (50×10×40 cm) mounted 50 cm above the floor. The floor of the arms was smooth. Light level was 30 lux. A mouse was placed on the central platform of the apparatus facing a closed arm. A camera on the ceiling of the test room was used to score and tape the animal's behavior from an adjacent room for a period of 7 min. Number of entries into open arms and time spent on open and closed arms were recorded. Arm entry was defined as all four feet in the arm. Animals were tested in a random order, and the maze was cleaned after each trial. Parameters were taken from 16 wild-type and 29 NEP-deficient mice. The person scoring the behavior was blind to the genetic constitution of the animals.

### Locomotor activity

Locomotor activity was monitored over a period of 15 min at 30 lx or 450 lx in a computerized activity meter (46×46 cm) with 15 photocells in each dimension (MOTITEST, TSE Bad Homburg, Germany). Travel distance and mean total activity (horizontal plus vertical) and time on the central field (25×25 cm) of 16 wild-type and 29 NEP-deficient mice were recorded. The person performing the test was unaware of the genetic constitution of the animals.

### Statistical analysis

Results are represented as mean±SEM. For determination of intergroup differences, Student's *t* test was used (InStat 2002, GraphPad, San Diego, USA). Significance was considered from a value of *P*<0.05.
